# Questionnaire-Based Everyday Reaction Time: Reliable, Valid, and Unobtrusive Measure of Cognition

**DOI:** 10.1093/geroni/igaa057.2015

**Published:** 2020-12-16

**Authors:** Nelson Roque, Martin Sliwinski, Nilam Ram

**Affiliations:** 1 Pennsylvania State University, University Park, Pennsylvania, United States; 2 Penn State University, University Park, Pennsylvania, United States

## Abstract

Experience sampling paradigms provide new opportunity for early identification of mild cognitive impairment (MCI). We investigated two research questions: (1) is time to complete a repeatedly administered survey (i.e., questionnaire-based everyday reaction time, q*bert) a reliable and valid measure of cognition? (2) does this measure distinguish MCI status? To answer these questions, we leveraged ecological momentary assessment (EMA) data from the Einstein Aging Study, where older adults (N=240) completed six daily surveys and cognitive assessments on smartphones over 14 days. Q*bert had good between-person reliability after two days (~11 EMAs) and excellent reliability from three to fourteen days. Q*bert moderately correlated with ambulatory cognitive measures of processing speed and memory binding (p’s < .001) and was significantly slower in those with MCI (p < .001). We propose q*bert as a reliable, valid, and unobtrusive measure of cognition when ambulatory cognitive assessments are not feasible.

